# Two crystal forms of a helix-rich fatty acid- and retinol-binding protein, Na-FAR-1, from the parasitic nematode *Necator americanus*

**DOI:** 10.1107/S1744309112023597

**Published:** 2012-06-28

**Authors:** Mads Gabrielsen, M. Florencia Rey-Burusco, Kate Griffiths, Andrew J. Roe, Alan Cooper, Brian O. Smith, Malcolm W. Kennedy, Betina Corsico

**Affiliations:** a Institute of Infection, Immunity and Inflammation, University of Glasgow, Glasgow, Scotland; b Instituto de Investifaciones Bioquimicas de La Plata, CONICET–UNLP, Facultad de Ciencias Medicas, Universidad Nacional de La Plata, La Plata, Argentina; c Insitute of Biodiversity, Animal Health and Comparative Medicine, University of Glasgow, Glasgow, Scotland; d School of Chemistry, University of Glasgow, Glasgow, Scotland; e Institute of Molecular, Cell and Systems Biology, University of Glasgow, Scotland

**Keywords:** fatty acid- and retinol-binding proteins, parasitic nematodes, *Necator americanus*, Na-FAR-1

## Abstract

Na-FAR-1 is an unusual α-helix-rich fatty acid- and retinol-binding protein from *Necator americanus*, a blood-feeding intestinal parasitic nematode of humans. It belongs to the FAR protein family, which is unique to nematodes; no structural information is available to date for FAR proteins from parasites. Crystals were obtained with two different morphologies that corresponded to different space groups. Crystal form 1 exhibited space group *P*432 (unit-cell parameters *a* = *b* = *c* = 120.80 Å, α = β = γ = 90°) and diffracted to 2.5 Å resolution, whereas crystal form 2 exhibited space group *F*23 (unit-cell parameters *a* = *b* = *c* = 240.38 Å, α = β = γ = 90°) and diffracted to 3.2 Å resolution. Crystal form 2 showed signs of significant twinning.

## Abstract 2

Na-FAR-1, a fatty acid- and retinol-binding protein, was expressed in bacteria, purified and crystallized. Crystals grew in two different morphologies under the same conditions.

## 1. Introduction

Fatty acid- and retinol-binding (FAR) proteins are a structurally novel class of ∼20 kDa lipid-binding proteins that are only found in nematodes. They belong to a family of proteins that exist in around seven different isoforms in each species and which are differentially produced at different life-cycle and developmental stages (Kennedy *et al.*, 1997 ▶; Garofalo, Kennedy *et al.*, 2003 ▶). They are hypothesized to play roles in host–parasite interaction and pathogenesis through the sequestration or delivery of pharmacologically or immunologically active small lipids, although there is still much to learn about their biological functions. Pertinent to this, FAR proteins have been found to be prominent components of the secretions of nematode parasites of humans, other animals and plants (Kennedy *et al.*, 1997 ▶; Suire *et al.*, 2001 ▶; Basavaraju *et al.*, 2003 ▶). Their structures are predicted to be rich in α-helices and they have no structural counterparts in other animal groups (Kennedy *et al.*, 1997 ▶; Basavaraju *et al.*, 2003 ▶). The crystal structure of one FAR protein, Ce-FAR-7, from the free-living nematode *Caenorhabditis elegans* has recently been reported (Jordanova *et al.*, 2009 ▶). However, according to sequence comparison this protein belongs to a separate group within the FAR protein family that differs from those secreted by parasitic nematodes into host tissues (Garofalo, Rowlinson *et al.*, 2003 ▶). FAR proteins, which are already used as diagnostic tools (Burbelo *et al.*, 2009 ▶), are attractive potential targets for drug or vaccine development to generate new antihelminthic controls.

*Necator americanus* is a blood-feeding nematode hookworm which causes anaemia and growth stunting, infecting 750 million people in tropical and subtropical areas with poor hygiene and economic conditions (Hotez *et al.*, 2004 ▶). Among the genes transcribed at high levels by *N. americanus*, one that encodes a FAR protein, Na-FAR-1, has been identified (Daub *et al.*, 2000 ▶).

Here, we report the bacterial expression of recombinant Na-FAR-­1, its purification and its crystallization in two different space groups: *P*432 and *F*23.

## 2. Materials and methods

### 2.1. Protein expression and purification

The Na-FAR-1 coding sequence was obtained from the Nematode Transcriptome Database (NEMBASE4; sequence ID NAC00128) and an encoding cDNA was chemically synthesized (GeneArt AG, Regensburg, Germany) with a polyhistidine-sequence affinity tag added at the N-terminus and cloned into pET11a expression vector. The plasmid was sequence-verified and transformed into *Escherichia coli* BL21 (DE3) cells. The bacteria were grown in LB medium at 310 K; protein production was induced with 1.0 m*M* IPTG and cells were harvested after 4 h. The cells were resuspended in 40 ml binding buffer (20 m*M* Tris–HCl pH 7.8, 500 m*M* NaCl, 5 m*M* imidazole) and lysed by sonication. The lysate was cleared by centrifugation. The supernatant was applied onto a nickel-affinity column (Novagen, Darmstadt, Germany) and washed in ten column volumes (CV) of binding buffer, followed by 6 CV wash buffer (20 m*M* Tris–HCl pH 7.8, 500 m*M* NaCl, 20 m*M* imidazole). The protein was eluted with 20 m*M* Tris–HCl pH 7.5, 500 m*M* NaCl, 250 m*M* imidazole over 6 CV. A second purification step was performed using size-exclusion chromatography (Superdex 75 HR 10/300; GE Healthcare, Little Chalfont, England). The final protein buffer was 20 m*M* Tris–HCl pH 7.5. The typical protein yield was around 30 mg of protein per litre of culture. The molecular mass of this recombinant Na-FAR-1 was calculated to be 18 776.4 Da, including the affinity tag, and comprised 170 residues in total.

### 2.2. Crystallization

The protein was concentrated to approximately 5 mg ml^−1^ and initial crystallization attempts were performed in 96-well sitting-drop plates using vapour diffusion and three commercially available crystallization screens. The protein solution was mixed with reservoir solution in a 1:1 ratio to give a final volume of 1 µl using a Cartesian Honeybee 81 (Genomic Solutions, Huntingdon, England) and the trays were stored at 293 K. Small crystals (approximately 20 × 20 × 20 µm) were observed in Cryo Screen II (Emerald BioSystems, USA) condition No. 18 [40% polyethylene glycol (PEG) 300, 100 m*M* phosphate–citrate pH 4.2]. Larger optimized crystals were grown in 24-well sitting-drop trays (Hampton Research) using drops set up with a 1:1 ratio of protein solution and optimized reservoir solution (38% PEG 300, 100 m*M* phosphate–citrate pH 4.2) to give a final drop volume of 3 µl. Crystals appeared within 10 d. The crystals were plunged directly into a stream of cooled gaseous nitrogen (100 K; Oxford Cryosystems, Oxford, England) without any further cryoprotection.

### 2.3. Data collection and processing

Data were collected from initial crystals at station I02 of Diamond Light Source (DLS; Didcot, Oxfordshire, England). Low-resolution diffraction data were observed to beyond 7 Å. Data were collected from optimized crystals at stations I03 and I04 of DLS and were collected over 180° with 1° oscillation at wavelengths of 0.9763 and 0.9796 Å, respectively. Data were processed with *MOSFLM* (Leslie & Powell, 2007 ▶) and were scaled in *SCALA* (Evans, 2006 ▶). The space groups were confirmed by *POINTLESS* (Evans, 2006 ▶). Twinning analysis was performed by analysing the output from *CTRUNCATE* (part of *SCALA*). All of these programs are part of the *CCP*4 suite of programs (Winn *et al.*, 2011 ▶).

## 3. Results

**Figure 1:**
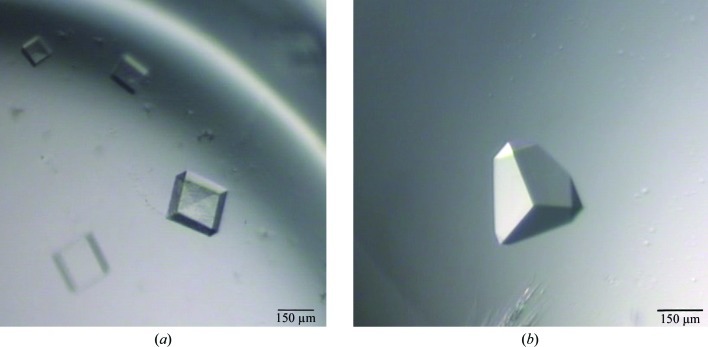
Crystals of Na-FAR-1 from *N. americanus*. The recombinant protein was purified from *E. coli* and optimized crystals were grown in 38% PEG 300, 100 m*M* phosphate–citrate pH 4.2. (*a*) Crystal form 1, space group *P*432 (*a* = 120.8 Å); (*b*) crystal form 2, space group *F*23 (*a* = 240.4 Å).

**Figure 2:**
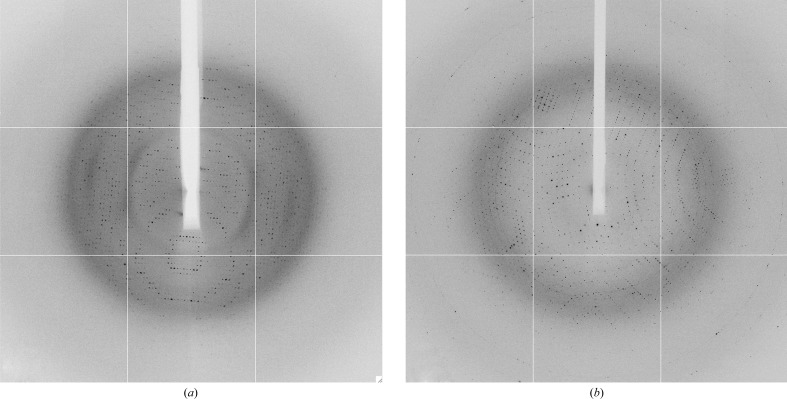
Sample diffraction patterns of crystal forms 1 (*a*) and 2 (*b*). Diffraction extended to beyond 2 Å resolution for form 1 and to beyond 2.5 Å resolution for form 2.

The crystals grew in two different morphologies with approximate dimensions of 200 × 200 × 100 µm (Fig. 1 ▶) in the same condition and in the same crystal tray well. Crystal form 1 (Fig. 1 ▶ *a*) belonged to space group *P*432 (unit-cell parameters *a* = *b* = *c* = 120.804 Å), with Bragg diffraction observed to beyond 2 Å resolution (Fig. 2 ▶ *a*). The data were cut back to 2.5 Å resolution based on an *R*_meas_ of 59.7% and an *R*_p.i.m._ of 11.1% in the highest resolution shell (see Table 1 ▶ for complete details).

**Figure 3:**
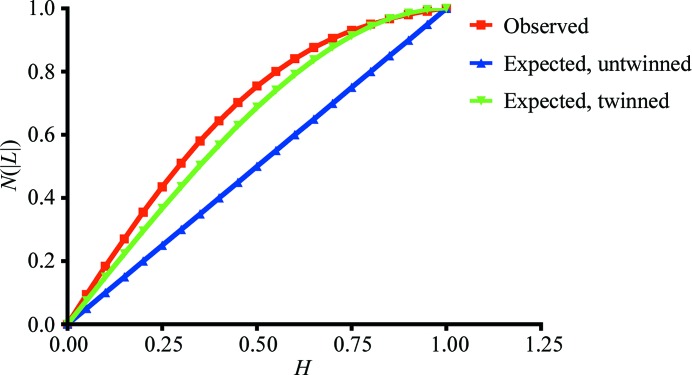
Output from *CTRUNCATE* for the *L*-test for twinning for crystal form 2. The observed values fitted closely to the values expected for perfect twinning.

Crystal form 2 (Fig. 1 ▶ *b*) was initially processed and scaled in space group *F*432 (unit-cell parameters *a* = *b* = *c* = 241.61 Å). However, inspection of the cumulative distribution of *L* (Fig. 3 ▶) and the moments of *E* (1.4 for the fourth moment; the expected values are 2 for an untwinned crystal and 1.5 for a perfect twin) suggested that the crystal was near-perfectly twinned and the actual space group was determined to be *F*23. The data were scaled to 3.2 Å resolution (Fig. 2 ▶ *b*) based on an *R*_meas_ of 53.0% and an *R*_p.i.m._ of 11.6% in the highest resolution shell (see Table 1 ▶ for full details).

Both crystal forms diffracted further, but the data were scaled to conservative estimates based on the *R*_meas_ and *R*_p.i.m._ values. Both data sets were anisotropic, with sectors of the crystals exhibiting poorer diffraction to a lower resolution, and these may give rise to the rapid increase in the *R*_meas_ and *R*_p.i.m._ values when the highest resolution limit is extended.

The nature of the asymmetric unit is unclear for both crystal forms, with crystal form 1 assumed to contain one or two subunits and crystal form 2 between four and eight subunits (see Table 2 ▶ for details). The corresponding Matthews coefficients range from 3.91 to 1.96 Å^3^ Da^−1^ for crystal form 1 and from 3.86 to 1.93 Å^3^ Da^−1^ for crystal form 2.

## 4. Conclusions

We have crystallized the fatty acid- and retinol-binding protein Na-FAR-1 from the parasitic nematode *N. americanus* in two crystal forms, one of which showed signs of significant twinning. The data set from crystal form 1 was scaled to 2.5 Å resolution, whereas the data set from crystal form 2 was scaled to 3.2 Å resolution. As there are no known structures with sufficiently high sequence similarity in the Protein Data Bank (Velankar *et al.*, 2012 ▶) to attempt molecular replacement, work is now under way to obtain experimental phases.

## Figures and Tables

**Table 1:** Data-collection and reduction statistics Values in parentheses are for the highest resolution shell. Diffraction was observed beyond the resolutions presented here, but the data were scaled to 2.50 Å for crystal form 1 and 3.20 Å for crystal form 2 based on the *R*_meas_ and *R*_p.i.m._ values. If the data were scaled further there was a dramatic increase in *R*_meas_ in particular. Crystal form 1 scaled to 2.1 Å resolution still showed 100% completeness and an 〈*I*/σ(*I*)〉 of 2.5 in the highest resolution shell (2.21–2.10 Å). However, the *R*_meas_ changed to 17.8% overall and 196.7% in the highest resolution shell. Crystal form 2 showed similar behaviour, although the data could only be scaled to 2.9 Å resolution with 100% completeness and an 〈*I*/σ(*I*)〉 of 3.0 in the highest resolution shell (3.06–2.90 Å), with an *R*_meas_ of 23.5% overall and 118% in the highest resolution shell.

	Crystal form 1	Crystal form 2
Space group	*P*432	*F*23
Unit-cell parameters (Å, °)	*a* = *b* = *c* = 120.80, α = β = γ = 90.0	*a* = *b* = *c* = 240.38, α = β = γ = 90.0
Resolution (Å)	49.32–2.50 (2.64–2.50)	69.39–3.20 (3.37–3.20)
Observed reflections	302052	404552
Unique reflections	10986	19069
Multiplicity	27.5 (28.5)	21.2 (20.9)
Completeness (%)	100.0 (100.0)	100.0 (100.0)
*R*_meas_ (%)	10.6 (59.7)	18.8 (53.0)
*R*_p.i.m._ (%)	2.0 (11.1)	4.1 (11.6)
〈*I*/σ(*I*)〉	33.3 (7.6)	18.3 (6.9)
Wilson *B* (Å^2^)	46.95	66.55

**Table 2:** The nature of the asymmetric unit has yet to be determined, but is expected to contain one or two subunits in the case of crystal form 1 and five or six subunits in the case of crystal form 2

Crystal form 1 (*P*432)			Crystal form 2 (*F*23)		
Matthews coefficient (Å^3^ Da^−1^)	Solvent content (%)	Monomers in asymmetric unit	Matthews coefficient (Å^3^ Da^−1^)	Solvent content (%)	Monomers in asymmetric unit
3.91	68.60	1	3.86	68.13	4
1.96	37.19	2	3.09	60.16	5
1.30	5.79	3	2.57	52.19	6
			2.20	44.22	7
			1.93	36.26	8
